# Knowledge and Attitudes Regarding Sickle Cell Anaemia and Genetic Testing Among Students of King Saud Bin Abdulaziz University for Health Sciences, Al‐Ahsa

**DOI:** 10.1155/nrp/6978487

**Published:** 2026-11-14

**Authors:** Amel Saied Mahmoud, Qonoot A. Almohammed, Lulu A. Alruhayman, Aryam A. Alrahib, Fajer M. Ghawwa, Wadha A. Almarri, Noura O. Alnami, Maryam S. Alsuwayj, Saja N. Alwadaani, Abdullah Mohammed Mutawa

**Affiliations:** ^1^ Medical-Surgical Nursing Department, Faculty of Nursing Cairo University Giza Egypt cu.edu.eg; ^2^ King Abdullah International Medical Research Center Riyadh Saudi Arabia kaimrc.med.sa; ^3^ Nursing Department College of Nursing King Saud Bin Abdul-Aziz University for Health Sciences Al Ahsa Saudi Arabia ksau-hs.edu.sa; ^4^ Ministry of the National Guard-Health Affairs Riyadh Saudi Arabia; ^5^ Pediatric Haematology King Abdulaziz Hospital Al Ahsa Saudi Arabia mngha.med.sa

**Keywords:** attitudes, genetic testing, knowledge, screening, sickle cell anaemia

## Abstract

**Background:**

Sickle cell anaemia (SCA) represents a significant global health challenge, especially in areas where the frequency of the sickle cell gene is high, as in Saudi Arabia. Millions of people globally suffer from this hereditary blood disorder. Genetic testing offers opportunities in prevention and personalized treatment approaches. Moreover, students at nursing and health colleges are the backbone of public education in the future. Hence, examining the levels of knowledge and attitudes regarding SCA and genetic testing among nursing and health sciences students has valuable implications for public health interventions, policy development, and community engagement initiatives.

**Aim:**

To assess the level of knowledge and attitudes regarding SCA and genetic testing among bachelor’s students at King Saud bin Abdulaziz University for Health Sciences, Al‐Ahsa, Saudi Arabia.

**Methods:**

This study employed a descriptive, cross‐sectional design using convenience sampling among 309 students at King Saud bin Abdulaziz University for Health Sciences in Al‐Ahsa. Data were gathered at a single time point via an online self‐reported survey including: (1) Demographic and background information form. (2) Participants’ knowledge assessment of SCA and genetic testing, which consisted of 13 items (score range 0–13). Scores ≥ 7.8 (≥ 60%) were classified as satisfactory; < 7.8 as unsatisfactory. (3) Participants’ attitudes towards SCA and genetic testing, which were measured on a 12‐item, 3‐point Likert scale (range 12–36). Scores ≥ 21.6 (≥ 60%) were positive; < 21.6 were negative.

**Results:**

The participants’ ages ranged from 17 to 23 years. Most of the participants were female (70.9%). Approximately 72.2% showed a satisfactory level of knowledge about SCA and genetic testing, with a total mean knowledge score (x̄ ± SD = 7.96 ± 2.527). The majority (90.6%) of the participants exhibited a positive attitude towards SCA and genetic testing, with a total mean attitude score (x̄ ± SD = 28.4 ± 4.6).

**Conclusion:**

Although this study demonstrated satisfactory knowledge and positive attitudes towards SCA and genetic testing, the findings are derived from a single university sample, constraining their broader generalizability.

**Recommendations:**

Future research should employ longitudinal designs to assess how knowledge and attitudes evolve and how they influence professional behaviour after graduation.

## 1. Introduction

Sickle cell anaemia (SCA), one of the most severe forms of sickle cell disease (SCD), is a major inherited haemoglobin disorder and a significant global public health concern because of its impact on quality of life, healthcare systems, and economic resources [1]. In 2021, approximately 7.74 million people were living with SCD worldwide, and 515,000 infants were born with SCD, including approximately 394,000 with the severe SS or Sβ^0^ genotypes associated with SCA [2]. In Saudi Arabia, SCA affects approximately 0.26% of the population, while about 4% carry the sickle cell trait; the prevalence is particularly high in the Eastern Province, where approximately 1.2% have SCA and 17% carry the trait [1]. More recent national newborn screening data identified an SCA (FS) pattern in 532 of 331,151 screened newborns (0.16%) between April 2023 and June 2024, with the highest numbers reported in Jazan, Al‐Ahsa, and the Eastern Province [3].

Despite advances in medical knowledge and therapeutic modalities, SCA remains a major global health concern associated with chronic pain, progressive organ damage, increased infection susceptibility, and reduced life expectancy [4, 5]. Effective management and prevention of SCA in Saudi Arabia may be challenged by social and cultural factors surrounding genetic disorders and premarital decision‐making. For instance, concerns about social stigma and the potential impact of genetic disorders on marriage decisions may influence perceptions of genetic testing and premarital screening [6]. Furthermore, knowledge gaps persist even among medical students, particularly regarding the management and some aspects of SCA [7].

In nursing and health sciences education, genetic testing extends beyond routine laboratory diagnostics; it encompasses a comprehensive clinical practice involving risk assessment, carrier identification, ethical support, and patient counselling. Therefore, healthcare professionals play a vital role in implementing early preventive strategies, including carrier screening and empowering individuals to make informed reproductive and health choices [8, 9]. Students enrolled in nursing and health sciences programs represent a key demographic situated at the intersection of academic training, community influence, and personal family planning. Developing clinical self‐confidence and educational satisfaction during their training is critical for preparing these trainees as competent clinicians and public health advocates [10, 11].

Assessing knowledge and attitudes regarding SCA and genetic testing requires examining both individual‐level determinants (e.g., health beliefs and personal knowledge) and organizational‐level determinants (e.g., structured genomic curriculum integration). While individual readiness is essential, a persistent gap remains in organizational support and standardized genetic training within health sciences education [12, 13]. However, the present study focuses specifically on individual‐level knowledge and attitudes towards SCA and genetic testing among health sciences students. Furthermore, framing health‐seeking behaviours through the lens of student agency positions health sciences trainees not as passive policy recipients, but as active, autonomous agents capable of advocating for ethical screening and driving informed decision‐making within their communities [14].

A comprehensive understanding of SCA—encompassing inheritance patterns, early diagnostic screening, and therapeutic management—empowers future healthcare professionals to make informed personal choices and provide accurate patient guidance [11]. Although evidence suggests that healthcare trainees often demonstrate satisfactory baseline knowledge of SCA, critical knowledge deficits persist in genetic susceptibility and premarital screening nuances [15]. For future health care providers, genomic competence extends beyond factual recall to include interpreting complex genetic risk, maintaining privacy, securing informed consent, and addressing ethical dilemmas [8]. However, genetics education remains inconsistently integrated across nursing and health sciences curricula [16, 17]. This gap is especially critical in Saudi Arabia, where the established Mandatory Premarital Screening Program and expanding precision health initiatives require a workforce skilled in genetic counselling and risk communication [18].

Recent evidence from Saudi Arabia and the Gulf region indicates persistent gaps in knowledge regarding SCD/SCA and its genetic implications. Studies among the Saudi population and university students have reported inadequate knowledge of SCD, inheritance, and premarital screening despite generally favourable attitudes towards screening [7, 19]. Similarly, evidence from the United Arab Emirates has demonstrated limited genetic knowledge alongside favourable attitudes towards genetic testing [20].

Consequently, this research attempted to guide focused educational efforts, healthcare policies, and interventions in line with and in concert with the evaluation of their care programs by providing a clearer current understanding and perceptions within this demographic. It elucidated this matter in the Kingdom of Saudi Arabia to contribute to the corpus of information regarding this field, aiming to reduce the burden of SCA and enhance the overall health outcomes of the community in Saudi Arabia. Therefore, the current study aimed to assess the level of knowledge and attitudes regarding SCA and genetic testing among bachelor’s students at King Saud bin Abdulaziz University for Health Sciences, Al‐Ahsa, Saudi Arabia.

## 2. Methods

### 2.1. Study Aim and Objective

This study aimed to assess the level of knowledge and attitudes regarding SCA and genetic testing among bachelor’s students at King Saud bin Abdulaziz University for Health Sciences, Al‐Ahsa, Saudi Arabia.

The primary hypothesis of the study was that the majority of students would demonstrate satisfactory knowledge and positive attitudes towards SCA and genetic testing.

A secondary hypothesis was that knowledge and attitudes would be positively correlated.

#### 2.1.1. Specific Objectives

- Evaluate the level of knowledge regarding SCA and genetic testing among bachelor’s students at King Saud bin Abdulaziz University for Health Sciences, Al‐Ahsa, Saudi Arabia.
- Assess the attitudes regarding SCA and genetic testing among King Saud bin Abdulaziz University for Health Sciences students in Al‐Ahsa, Saudi Arabia.

#### 2.1.2. Secondary Objectives

- Investigate the correlation between students’ knowledge and attitudes regarding SCA and genetic testing within the sample studied.

### 2.2. Design and Setting

This study employed a descriptive, cross‐sectional design, which is the most appropriate framework for an exploratory baseline assessment. The study was conducted through an online self‐reported survey at King Saud bin Abdulaziz University for Health Sciences (KSAU‐HS), Al‐Ahsa. This involved the University Pre‐Professional Program (UPPP), which provides foundational sciences and English courses before students enter their professional colleges: Colleges of Nursing for females only, and Applied Medical Sciences for females and males. The latter includes many programs, including Clinical Nutrition, Emergency Medical Services, Occupational Therapy, Radiological Sciences, Clinical Laboratory Sciences, and Respiratory Therapy.

### 2.3. Study Subjects

The study targeted undergraduate students enrolled at the time of data collection, conducted between August and November 2024 during the academic year (2024–2025), Fall semester. Inclusion criteria: all university students, regardless of level and speciality. Exclusion criteria: those who were unwilling to take part.

#### 2.3.1. Sample Size and Sampling Technique

In the current study, convenience sampling based on voluntary response was employed to target university students; this method allowed for efficient access to a large pool of potential respondents among university students. However, recruitment of participants used standardized, broad distribution across academic levels without positioning the study as a test of SCA, and genetic knowledge, thereby minimizing the potential for self‐selection based on perceived knowledge. The target population comprised 1558 eligible students at the study site. The minimum required sample size for the survey was determined using the Raosoft (n.d.) sample size calculator, based on a 95% confidence level, a 5% margin of error, and a 50% response distribution. The calculation is based on Cochran’s sample size formula for estimating a population proportion:

*n*_0_ = *z* ^2^ *·* *p*(1 − *p*)/*e* ^2^. With *z* = 1.960, *p* = 0.5, *e* = 0.050 ⟶ *n*_0_ = 384.1.

Finite population correction applied: *n* = *n*_0_/(1 + (*n*_0_ − 1)/*N*) with *N* = 1558 ⟶ *n* = 309.

In addition, an a priori power analysis was conducted using G∗Power software (version 3.1.9.7) for the secondary inferential analysis, namely, the two‐tailed Pearson correlation between knowledge and attitude scores. Assuming a medium correlation effect size of (*r* = 0.30), a significance level of (*α* = 0.05), and statistical power of 80%, the required sample size was approximately 84 participants. The final sample of 309 participants exceeded the sample requirement for the planned correlation analysis. To ensure data integrity, incomplete responses or those without informed consent were excluded. Data collection remained active until the predetermined stopping criterion of 309 fully completed, valid responses was successfully reached, at which point the survey link was closed for final statistical analysis.

### 2.4. Instruments

Data were gathered through an online 30‐item questionnaire modified from existing studies developed by the researchers after an extensive literature review [21, 22]. The survey consisted of three sections:

- Demographic and background information form: included five questions such as age, gender, marital status, academic level, and speciality.
- Knowledge assessment of SCA and genetic testing. It had two parts with 13 multiple‐choice questions. The first part encompassed 8 questions related to knowledge about SCA, while the second part had 5 questions regarding knowledge concerning genetic testing. Questions were scored with one point for correct responses and 0 points for incorrect responses. The maximum possible score for each participant was 13, while the least was zero.
 Scoring: A score of 7.8 or higher (≥ 60%) was considered a satisfactory level of knowledge, whereas a score of less than 7.8 (< 60%) was classified as an unsatisfactory level of knowledge. Although the literature often utilizes a 50% cut‐off to define poor knowledge [23], researchers selected a 60% threshold to align with university academic passing standards in health sciences and established genomic literacy research [9]. This provides a meaningful boundary between basic foundational literacy and significant knowledge deficits.
- Participants’ attitudes towards SCA and genetic testing (12 items). This is subsequently divided into two sections. The first section held 4 questions related to the attitudes of the participants regarding SCA. The second section had 8 questions regarding attitudes towards genetic testing.
 The responses to the attitude questions were scored on a three‐point Likert scale with options ranging from 1 (disagree), 2 (neutral), and 3 (agree).
 Scoring: a score of less than 21.6 (< 60%) was classified as a negative attitude, whereas a score of ≥ 21.6 (≥ 60%) was considered a positive attitude. Items 1, 5, and 11 were scored inversely.
 The questionnaire was reviewed by a panel of experts, including four faculty members and a physician with experience in SCA and genetics. In the current study, knowledge assessment and participants’ attitudes towards SCA and genetic testing demonstrated Cronbach’s alpha of 0.80 and 0.87, respectively, indicating good internal consistency.
 A pilot study was carried out on 10% (*n* = 31) of the sample before the main data collection to check the clarity and reliability of the questionnaire. Based on these findings, minor revisions were made before using the final version of the tool. The pilot participants were excluded from the main study and final statistical analysis to ensure that all participants were assessed using the final version of the instrument.

### 2.5. Procedure

This research underwent two phases. Phase one, the preparation phase, involved designing the tool for data collection and obtaining necessary approvals, including IRB and approval from the university administration. Phase two, data collection phase conducted between August and November 2024 during the Fall semester of the 2024‐2025 academic year, by conducting an online self‐administered survey. The study questionnaire link was sent through formal mail and WhatsApp to the participants.

The first section of the questionnaire was an electronic form of consent with an overview of the study, showing the contact information of the principal investigator and an agreement button to participate. A Google Form was used for this survey, which required respondents to respond to each question individually before they moved on to the next question. To reduce missing data, all questions needed to be addressed. To improve the response rate, reminders were sent to encourage participation in filling out the survey. Additionally, arrangements for meeting the students in their classes were made in collaboration with the academic affairs in each college.

### 2.6. Statistical Analysis

Collected data were tabulated, computed, and analysed using IBM SPSS Statistics for Windows (version 29.0; IBM Corp., Armonk, NY, USA) [24]. Descriptive statistics were summarized using means and standard deviations for continuous numerical data, while categorical variables were expressed as frequencies and percentages. Bivariate relationships were evaluated using Pearson’s correlation analysis, with effect sizes interpreted according to standard thresholds: *r* = 0.10–0.29 (small), *r* = 0.30–0.49 (moderate), and *r* ≥ 0.50 (large). All statistical tests were two‐tailed, and significance was established at *p* ≤ 0.05. Given the exploratory nature of the study, no adjustments for multiple comparisons were applied; therefore, findings with *p*‐values between 0.01 and 0.05 should be interpreted with appropriate caution. Exact 95% confidence intervals (CI) for key correlations were computed as follows: total knowledge versus total attitude (*r* = 0.587, 95% CI [0.51, 0.66]); knowledge about SCA versus attitude towards SCA (*r* = 0.327, 95% CI [0.22, 0.43]); and knowledge about genetic testing versus attitude towards genetic testing (*r* = 0.607, 95% CI [0.53, 0.68]).

## 3. Ethical Considerations

Approvals were obtained from the research committee—College of Nursing, Al‐Ahsa, Saudi Arabia; the IRB was obtained from King Abdullah International Medical Research Centre (KAIMRC) (Date: 21 August 2024, Identification number: IRB/1195/24), and the university administration. The purpose of the study was explained at the beginning of the online survey to the participants. Participants’ autonomy was ensured, and their involvement was completely voluntary. Informed consent was obtained from the respondents who decided to take part in the research study. The anonymity and confidentiality of participants were guaranteed by coding. The responses will not be available for unauthorized access. The research was noninvasive, with no intervention or alteration of human beings, and participants were not subject to any bodily or psychological harm.

## 4. Results

Table 1 depicts the characteristics of the study sample. The age ranged from 17 to 23 years, with a mean of 19.83 ± 1.435 years. The majority (70.9%) of the sample were females, and the vast majority (93.5%) were single. About one‐third (33.3%) and nearly half (48.2%) of the study sample were fourth‐year and nursing students, respectively.

**TABLE 1:** Demographic characteristics of the students studied (*n* = 309).

Characteristics	*N*	%
Age (in years)		
(17–< 20)	124	40.1
(20–24)	185	59.9
(Range) mean ± SD	**(17–23) 19.83 ± 1.435**	
Gender		
Male	90	29.1
Female	219	70.9
Marital status		
Single	289	93.5
Married	18	5.8
Divorced	1	0.3
Widow	1	0.3
Academic year		
First	88	28.5
Second	55	17.8
Third	63	20.4
Fourth	103	33.3
Speciality		
Clinical laboratory	19	6.1
Clinical nutrition	15	4.9
Emergency medical services	33	10.7
Nursing	149	48.2
Occupational therapy	16	5.2
Radiology	17	5.5
Respiratory therapy	60	19.4

*Note:* Percentages may not sum to 100 due to rounding. Bold formatting indicates the age range, mean, and SD.

Table 2 depicts that a substantial majority (83.8%) of students demonstrated satisfactory knowledge of SCA, with a mean knowledge score of 4.91 ± 1.413. Approximately three‐fifths (61.5%) of the surveyed students exhibited satisfactory knowledge of genetic testing, with a mean score of 3.05 ± 1.60. Overall, the majority (72.2%) of students displayed satisfactory total knowledge levels (mean ± SD = 7.96 ± 2.527).

**TABLE 2:** Knowledge of SCA and genetic testing among health sciences students (*n* = 309).

Knowledge domains	Unsatisfactory		Satisfactory		Range	X ± SD
	*N*	%	*N*	%		
Knowledge about SCA	50	16.2	259	83.8	(1–8)	4.91 ± 1.413
Knowledge about genetic testing	119	38.5	190	61.5	(0–5)	3.05 ± 1.601
Total knowledge level	86	27.8	223	72.2	(2–13)	7.96 ± 2.527

*Note:* (< 60%) unsatisfactory (≥ 60%) satisfactory.

Table 3 shows that more than half (57.6%) of health science students would terminate a relationship if there was a risk of having a child with SCA, indicating strong influence of genetic risk on personal decisions. Over half (54.4%) of the students reported having no issue working with a sickle‐cell patient. Additionally, a considerable proportion (65.7%) reflects that the students would educate their family and others about the risks of SCA. More than half (57.3%) expressed empathy towards individuals living with SCA. Furthermore, the same table presents a positive attitude among health sciences students towards genetic testing. About half (46.9%) believe genetic testing could lead to marriage denial, and 61.8% disagree that it does more harm than good. Additionally, 48.2% encourage nonconsanguineous marriage, while 38.2% are neutral on choosing a partner based on blood genotype. A majority supports mandatory premarital genetic testing (66.7%) and is open to premarital counselling (59.9%). Moreover, 43.7% are neutral on concerns about stigmatization, and 54.4% agree that a pregnant woman has the right to screen her foetus.

**TABLE 3:** Attitudes towards SCA and genetic testing among health sciences students (*n* = 309).

Variables	Disagree		Neutral		Agree	
	*N*	%	*N*	%	*N*	%
*Attitudes towards SCA*						
1. Willing to continue with a relationship despite the risk of having offspring with SCA.	178	57.6	91	29.4	40	12.9
2. I have no issue working with a sickle‐cell patient	39	12.6	102	33.0	168	54.4
3. I will educate my family and others about risks of SCA.	42	13.6	64	20.7	203	65.7
4. I feel empathy towards individuals living with SCA.	52	16.8	80	25.9	177	57.3

*Attitudes towards genetic testing*						
5. Genetic testing may lead to denial of marriage for a couple	58	18.8	106	34.3	145	46.9
6. Genetic testing will do more harm than good for society	191	61.8	61	19.7	57	18.4
7. Encourage non‐consanguineous marriage (non‐relatives’ marriage)	55	17.8	105	34.0	149	48.2
8. The choice of my partner will be dependent on his/her blood genotype	83	26.9	118	38.2	108	35.0
9. I prefer that genetic testing to be mandatory before marriage	33	10.7	70	22.7	206	66.7
10. Willing to do premarital genetic counselling and screening	41	13.3	83	26.9	185	59.9
11. Genetic testing may lead to stigmatization of the person if diagnosed positive	90	29.1	135	43.7	84	27.2
12. A pregnant woman has the right to have her foetus screened for the risk of having SCA	49	15.9	92	29.8	168	54.4

Table 4 clarifies that the study sample’s overall attitude scores ranged from 12 to 36, with a total mean score of 28.4 ± 4.46. The plurality (90.6%) had a positive attitude towards SCA and genetic testing.

**TABLE 4:** Total attitude scores regarding SCA and genetic testing among health sciences students (*n* = 309).

Attitude domains	Negative		Positive		Range	X ± SD
	*N*	%	*N*	%		
Students’ Attitudes towards SCA	22	7.1	287	92.9	(4–12)	9.79 ± 1.88
Students’ Attitudes towards Genetic Testing	24	7.8	285	92.2	(8–24)	18.53 ± 3.15
Student’s total attitude score (SCA + genetic testing)	29	9.4	280	90.6	(12–36)	28.4 ± 4.6

*Note:* (< 60%) negative (≥ 60%) positive.

Table 5 shows that there is a statistically significant positive relationship between the student’s knowledge and attitude towards SCA (*r* = 0.327, *p < 0.001*). Similarly, there is a significant positive relation between knowledge about genetic testing and attitude towards genetic testing (*r* = 0.607, *p* < 0.001). Overall, there is a statistically significant positive relation between the total knowledge scores and the total attitude score: (*r* = 0.587, *p* < 0.001). The results suggest that students with greater knowledge in these domains create a more positive attitude.

**TABLE 5:** Correlation between students’ knowledge and attitude regarding sickle cell anaemia and genetic testing among the sample studied (*n* = 309).

Variables	*r*	*p*‐value
Students’ knowledge about SCA × student’s attitudes towards SCA.	0.327	0.000^∗∗^
Students’ knowledge about genetic testing × student’s attitudes towards genetic testing.	0.607	0.000^∗∗^
Students’ total knowledge score about SCA × student’s total attitude score towards SCA	0.587	0.000^∗∗^

^∗∗^Statistically high significance at level *p < 0.001*.

## 5. Discussion

This study evaluated the level of knowledge and attitudes regarding SCA and genetic testing among undergraduate health sciences students at King Saud bin Abdulaziz University for Health Sciences in Al‐Ahsa, Saudi Arabia. Overall, the findings reveal a high baseline of scientific awareness and positive attitudes towards genetic testing within this cohort. Specifically, 72.2% and 90.6% of participants demonstrated satisfactory knowledge levels and positive attitudes towards SCA and genetic testing.

The demographic data of the study indicate that the majority of participants were between the ages of 20 and 23, representing 59.9% of the sample. The sample was predominantly female, constituting 70.9% of the participants. This could be related to the fact that the number of female students at the university exceeds that of male students. In terms of marital status, singles had the highest percentage (93.5%). This reflects the focus on academic and career goals at this stage of life. Fourth‐year students constituted the largest group, constituting 33.3% of the sample. Nursing was the most predominant major, accounting for 48.2% of participants enrolled in the program. This could be attributed to the fact that the nursing major has more students than the applied medical science majors (clinical laboratories, clinical nutrition, emergency medical services, occupational therapy, radiology, and respiratory therapy).

The relatively high proportion of students achieving satisfactory knowledge (72.2%) may reflect multiple factors, including the impact of structured health education, public health dissemination, and national initiatives under the Health Sector Transformation Program of Saudi Vision 2030 [25]. Additionally, health sciences students’ access to digital health resources further supports their understanding of SCA and available screening modalities, where integrating innovative educational tools relies heavily on socio‐technical readiness and institutional trust to foster sustainable learning adoption [26]. However, because prior exposure to noncurricular awareness campaigns was not measured, a potential ceiling effect should be considered when interpreting these high knowledge scores.

These results align with findings by Agbozo et al. [15], who observed high knowledge levels and favourable attitudes among healthcare trainees exposed to targeted health awareness initiatives. Conversely, Zakariyah et al. [9] identified notable knowledge gaps among medical trainees in Saudi Arabia, with over 60% demonstrating slight knowledge of core genetic principles. Substantial knowledge deficits have also been documented among non–health cohorts, such as university students in Uganda [23] and Kenyan youth [27], highlighting gaps in knowledge of SCDs and its inheritance among young adults.

The results of this study offer important insights into the attitudes of nursing and health sciences students towards SCA and genetic testing. A substantial majority, 90.6%, demonstrated a positive attitude towards these topics, proposing high acceptance of preventive genetic services. This may be related, in part, to the education of the health sciences students, which encompasses biomedical concepts and awareness of the prevalence of haemoglobinopathies in Saudi Arabia, a country characterized by high rates of consanguinity. However, these explanatory factors were not directly measured in the present study. Previous research has demonstrated that needs‐based education can improve knowledge and attitudes towards medical genetics among medical students [11].

Furthermore, the results showed a significant positive correlation between students’ knowledge and attitudes, suggesting that higher levels of knowledge were associated with more favourable attitudes towards SCA and genetic screening. The finding may be understood through the lens of the Health Belief Model (HBM), particularly its key constructs of perceived susceptibility, perceived severity, perceived benefits, and perceived barriers [28]. Greater knowledge may help students recognize their potential susceptibility to carrying the genetic trait, understand the severity of SCA and its complications, and appreciate the perceived benefits of genetic testing as a preventive health behaviour. Conversely, knowledge gaps may contribute to greater perceived barriers or uncertainty regarding screening.

This finding is consistent with previous research among Saudi adults, which reported a significant correlation between knowledge and attitudes towards SCD and premarital genetic counselling [21]. Similarly, a study among university students in Ghana, which reported a weak but statistically significant positive correlation between knowledge and attitudes towards SCD (*r* = 0.25, *p* < 0.001), suggesting that higher levels of knowledge were associated with more positive attitudes [29]. Nevertheless, given the cross‐sectional design of the present study, the observed association should not be interpreted as evidence of a causal relationship between knowledge and attitudes.

In contrast, Kotb et al. [30] found in an intervention study among secondary school students in the Jazan region of Saudi Arabia a significant improvement in knowledge following a health education program, whereas attitudes towards SCA and premarital screening did not change significantly. The authors suggested that attitudinal change may be influenced by factors beyond knowledge, including perceived seriousness and susceptibility to the disease, perceived benefits and barriers, and cultural practices that may hinder changes in attitudes. These findings highlight that improving knowledge may be necessary but not sufficient to produce corresponding changes in attitudes.

This discrepancy partly reflects the influence of cultural and belief systems on how knowledge is internalized and translated into health‐related decisions. Evidence from Saudi university students suggests that attitudes towards consanguineous marriage and genetic testing are shaped not only by knowledge but also by cultural values, family traditions, and social expectations [31]. Similarly, a qualitative study indicates that cultural beliefs, religious values, and social pressures can influence decisions regarding premarital and genetic testing [32]. Thus, knowledge may contribute to more favourable attitudes towards SCA and genetic screening, but its influence may be moderated by broader cultural and social norms.

Overall, the satisfactory knowledge and highly favourable attitudes identified in this study provide a strong foundation for empowering health sciences students as clinical advocates and health educators. To sustain and build upon these positive outcomes, health sciences programs must continually adapt their instructional strategies to align with regional health priorities and educational constraints. Integrating such context‐tailored educational approaches ensures that health sciences and nursing training directly address local epidemiological burdens, such as haemoglobinopathies in endemic regions [33]. While this study analysed health sciences students as a unified cohort, future research should evaluate nursing and applied medical sciences disciplines separately to account for differences in clinical exposure and identify tailored educational needs.

## 6. Limitation

Several methodological limitations should be acknowledged. First, the cross‐sectional design prevents causal inferences. Second, the sample was limited to health sciences students at a single university (KSAU HS, Al Ahsa), and 70.9% were female, limiting generalizability. Third, the convenience sampling method may have introduced selection bias. Fourth, the study relied on self‐reported attitudes, which may be subject to social desirability bias. Fifth, because some data were collected during class time, students with higher attendance may be overrepresented, potentially biasing results towards higher knowledge scores. Finally, while the overall sample size was sufficient for primary analyses, nonsignificant subanalyses may be subject to power constraints.

## 7. Conclusion

In brief, this exploratory cross‐sectional study provides baseline insights into the knowledge and attitudes towards SCA and genetic testing among health sciences students at a single academic institution in Saudi Arabia. The findings demonstrate a generally satisfactory baseline knowledge paired with overwhelmingly positive attitudes towards SCA and genetic testing. Given the single‐centre design and female‐majority sample, these results should be interpreted as exploratory rather than broadly generalizable across all regional health cohorts. Nevertheless, as future healthcare providers, health sciences trainees bear a professional and ethical responsibility to disseminate accurate information regarding SCA, support informed patient decision‐making towards genetic testing, and combat sociocultural stigma surrounding hereditary blood disorders. Integrating culturally grounded education and experiential learning enables students to translate positive baseline attitudes into proactive preventive health practices.

## 8. Recommendation

The following recommendations are suggested:

### 8.1. Practical, Educational, and Policy Implications

1. Academic Leadership & Curriculum Committees (Systemic & Educational Level):
  • Academic Institutions and curriculum committees can enrich existing health sciences programs by embedding a competency‐based module on haemoglobinopathies.
  • Curricula can utilize interactive case‐based learning scenarios focused on premarital and prenatal genetic testing, complemented by guest sessions featuring genetic counsellors. This can consolidate students’ technical confidence and foster supportive attitudes.
  • Integrating these modules through blended learning formats offers a cost‐effective, operationally feasible, and easily adaptable strategy within existing infrastructure.
  • Implementation should be guided by established change management models (such as Rogers’ Diffusion of Innovations) to foster institutional readiness and sustainable adoption.
2. Healthcare Policymakers and Leadership (Policy & Professional Level)
  • Healthcare policymakers and university leadership can establish formal channels to align classroom instruction directly with the Saudi National Premarital Screening Framework.
  • Educational programs must explicitly link such modules to health sciences codes of ethics, emphasizing the practitioner’s moral obligation to uphold patient autonomy, confidentiality, and informed decision‐making in reproductive health.
  • Universities can leverage campus digital platforms and student‐led health organizations to run student‐led awareness campaigns, bridging the gap between national public health policy and university‐level health advocacy.

### 8.2. Future Research Directions

To build upon these preliminary findings and address the limitations of single‐centre cross‐sectional designs, future investigations should pursue the following targeted research avenues:

• Employ multicentre longitudinal tracking to evaluate how SCA knowledge, ethical reasoning, and attitudes evolve chronologically from academic admission through clinical internships to graduation.
• Conduct stratified comparative studies to examine whether knowledge and attitudes differ significantly between academic tracks (e.g., Nursing vs. Applied Medical Sciences) and across different academic year levels (e.g., junior vs. senior cohorts).
• Expand sampling to include non–health science university disciplines and the public to assess baseline population literacy.

## Author Contributions

Amel Saied Mahmoud: writing–review and editing, writing–original draft, resources, methodology, investigation, instrument review, conceptualization and data collection. Qonoot A. Almohammed: writing–review and data collection. Lulu A. Alruhayman: writing and data collection. Aryam A. Alrahib: writing and data collection. Fajer M. Ghawwa: writing, methodology and data collection. Wadha A. Almarri: writing–review and data collection. Noura O. Alnami: writing–review and data collection. Maryam S. Alsuwayj: writing–review and data collection. Saja N. Alwadaani: writing–review and data collection. Abdullah Mohammed Mutawa: instrument review.

## Funding

 This research did not receive any specific grant from funding agencies in the public, commercial, or not‐for‐profit sectors.

## Ethics Statement

This study was approved by the Institutional Review Board of King Abdullah International Medical Research Center (KAIMRC) (Date: 21 August 2024, Identification number: IRB/1195/24). All participants were informed that participation was voluntary and anonymous.

## Consent

Informed consent was obtained from all participants at the beginning of the questionnaire.

## Conflicts of Interest

The authors declare no conflicts of interest.

## Data Availability

The data that support the findings of this study are available upon request from the corresponding author. The data are not publicly available due to privacy or ethical restrictions.
